# Developing a Curriculum for Trauma and Orthopaedic Higher Surgical Trainees: Implementing Small Group Teaching in a Regional Programme

**DOI:** 10.7759/cureus.73936

**Published:** 2024-11-18

**Authors:** Lucy C Walker, Joe Barrett-Lee, Alex Chowdhury, Adam C Stoneham, Evan Davies, Daniel Marsland

**Affiliations:** 1 Trauma and Orthopaedics, Hampshire Hospitals NHS Foundation Trust, Basingstoke, GBR; 2 Trauma and Orthopaedics, Queen Alexandra Hospital, Portsmouth, GBR; 3 Trauma and Orthopaedics, University of Otago, Christchurch, NZL; 4 Trauma and Orthopaedics, North Shore Hospital, Auckland, NZL; 5 Trauma and Orthopaedics, University Hospital Southampton NHS Foundation Trust, Southampton, GBR

**Keywords:** didactic lectures, frcs preparation, orthopaedic registrar training, small group teaching, trainee feedback

## Abstract

Introduction

Small group teaching strategies have been considered more efficient than didactic lectures in motivating trainees. There is a lack of studies focusing on the use of small group discussions for higher surgical training. The aims of the current study were to report the implementation of small group teaching and to move away from didactic lectures for a regional trauma and orthopaedics higher surgical trainee teaching programme.

Methods

Feedback from the teaching sessions was prospectively collected from trainees regarding the delivery of their surgical curriculum and meeting their training needs using formal feedback forms. Twenty-two consecutive regional teaching sessions were included in the analysis, twelve using a variety of teaching methods including didactic lectures, and ten sessions aiming to focus entirely on a small group format. Comparisons were then made between the two teaching formats.

Results

A weak correlation was found between the teaching feedback scores and the amount of the session comprised of the small group format as per the published schedules (rs = 0.296) and as rated by the trainees (rs = 0.061). There was a statistically significant positive correlation between the proportion of small group format teaching and how useful the trainees rated this for their education (rs = 0.597, p = 0.005).

Conclusions

The assessment of small group format teaching in higher surgical trainee education has not been previously reported. The current study shows that trauma and orthopaedics higher surgical trainee satisfaction with Fellowship of Royal College of Surgeons (FRCS) curriculum teaching has a significant correlation with a greater proportion of small group format.

## Introduction

The Joint Committee for Surgical Training (JCST) regulations for higher surgical training include a formal teaching programme to be coordinated and run by each deanery [1]. The traditional teaching method is lecturing, which has been frequently used despite numerous identified pitfalls [2]. It has been reported that due to minimal student participation in didactic lectures, which often have a teacher-centred and monotonous format, students retain only 10-20% of the information given [3].

Student-centred teaching strategies, such as small group discussions, have been considered more effective than lecture-based methods at motivating and encouraging students to realize their potential [4]. In surgical training, the use of small group teaching has shown positive results in the teaching of anatomy when compared to didactic lectures [5]. Specifically in trauma and orthopaedics, small group teaching has been positively received by medical students [6, 7]. However, with regards to post-graduate surgical trainees, although some evidence assesses the use of different teaching modalities such as virtual teaching [8, 9], no studies exist focusing on the use of small group discussions for the higher surgical training curriculum.

The trauma and orthopaedic higher surgical trainee teaching programme within the authors’ NHS deanery runs a whole-day session on a fortnightly basis for all pre-Fellowship of the Royal College of Surgeons (FRCS) orthopaedic registrars with a national training number. Based on the reported positive results of small group teaching on learning and trainee satisfaction in other areas of medical and surgical training [5, 10, 11], the programme transitioned from didactic lectures to this format of delivering training. Given the lack of literature assessing the use of small group teaching in post-graduate trauma and orthopaedics, the authors formulated this study. The objectives were to determine whether changing from didactic lectures to a small group discussion format for regional trauma and orthopaedic higher surgical training teaching sessions led to a change in trainee satisfaction with regards to meeting their training needs, as determined by their anonymised feedback. It was hypothesised that satisfaction rates would be higher for small group teaching compared with didactic teaching sessions.

## Materials and methods

From April 2023, the Wessex Deanery trauma and orthopaedics higher surgical trainee teaching programme format transitioned from didactic lectures to a greater focus on small group sessions, including case-based discussions, viva-style questioning, and clinical examinations with patients. The teaching sessions were delivered by a variety of trauma and orthopaedic trainers at the senior registrar, fellow, or consultant level, each with a broad spectrum of sub-specialty interests. All trainers were given guidance prior to their session on the format of teaching they should use.

Anonymized feedback following 22 all-day teaching sessions was prospectively collected via an online questionnaire. After each session, a link to the feedback form was emailed to all trainees, including both linear scale rating questions and free-text short answers. An example of the feedback questionnaire format is shown in Appendix 1. From April to December 2023, ten all-day teaching sessions were conducted with a greater focus on the small group teaching format. For comparison, feedback from the twelve teaching sessions conducted from September 2022 to March 2023 was also collected.

Data were collected on the number of trainees attending, grade of training (ST3-ST8), and the percentage of trainees who provided feedback. The mean feedback score was calculated for each day and converted into a percentage value. The percentage of each teaching session that comprised small group format was recorded based on the published schedules for each day. From April to December 2023, trainees also provided feedback on what percentage of the day they felt was interactive and then were asked to rate how useful this was compared to didactic lectures.

Comparisons were then drawn between the feedback from the small group-focused teaching sessions (April to December 2023) and those using a mixed format, including didactic lectures (September 2022 to March 2023). The data were also analyzed for correlations between the proportion of non-didactic teaching and the trainees' rating of the session. Conclusions regarding the usefulness of small group sessions in trauma and orthopaedics higher surgical training were then drawn.

Statistical analysis

A Shapiro-Wilk test indicated non-normally distributed data. A Mann-Whitney U test was used to compare linear variables between groups. Spearman’s Rho calculator was used to assess for correlation (weak 0.1-0.3, moderate 0.4-0.6, strong 0.7-1.0 [12]) between linear variables. A p-value of <0.05 was defined as statistically significant.

Ethical declaration

The authors conducted a prospective service evaluation; there was no additional patient contact and no requirement for formal ethical approval. The project was registered with the institutions’ audit departments and conducted in accordance with the Declaration of Helsinki and the guidelines for good clinical practice.

## Results

The 22 teaching sessions were included in the final analysis. The mean attendance was 26 trainees for the mixed format teaching and 23 for the small group format (p = 0.447). Table 1 shows the proportion of each training grade for both teaching formats. There was no significant difference in the mean percentage of trainees that provided feedback for the small group teaching compared to the mixed teaching, 56.4% and 45.7% respectively, p = 0.06 (Mann-Whitney U test).

**Table 1:** Attendees' stages of training for mixed and small group format teaching sessions. Statistical analysis with Mann-Whitney U test. ST: Specialty Training; T&O: Trauma & Orthopaedics.

	Percentage mean of number of attendees			
T&O Registrar level	Mixed format teaching	Small group teaching	U value	P-value (Mann-Whitney U test)
ST3-4	46.88	43.74	50.5	0.552
ST5-6	36.17	39.8	38.5	0.167
ST7+	17.58	16.45	54.5	0.741

**Figure 1:**
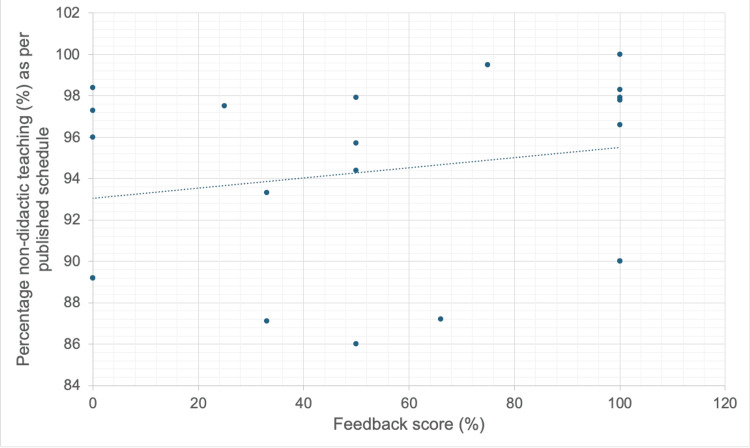
Correlation between feedback score and the percentage of non-didactic teaching on the published schedule.

Reported satisfaction rates were high for both styles of teaching. The mean feedback score percentage was not significantly different between the small group and mixed teaching formats, 94.6% and 94.3% respectively, p = 0.741 (Mann-Whitney U test). However, a positive correlation was found between a greater proportion of small group teaching and satisfaction score, rs = 0.296 (Spearman’s Rho calculator), but this did not reach statistical significance, p = 0.18 (Figure 1).

**Figure 2:**
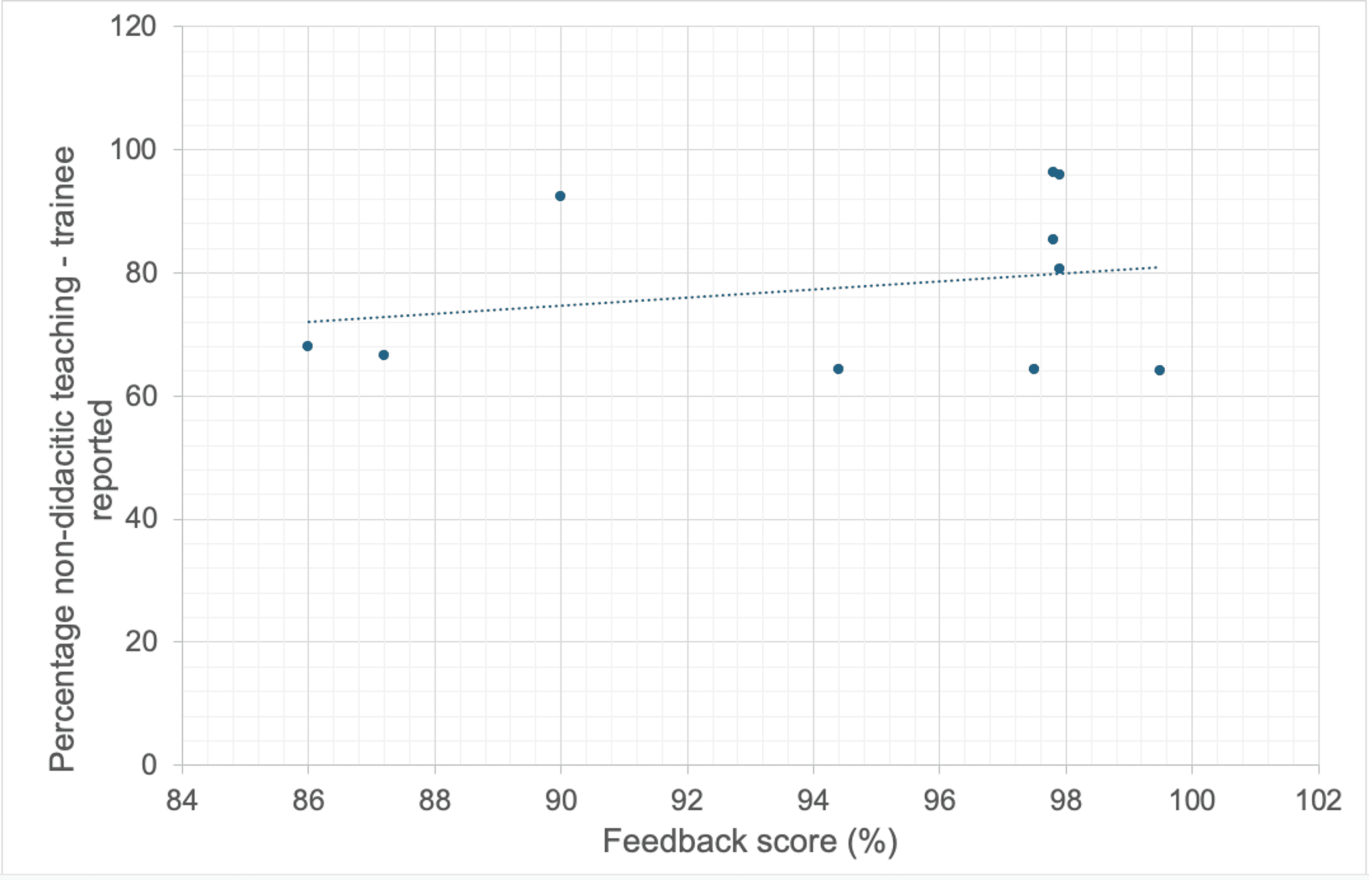
Correlation between feedback score and percentage of non-didactic teaching as per trainee ratings.

**Figure 3:**
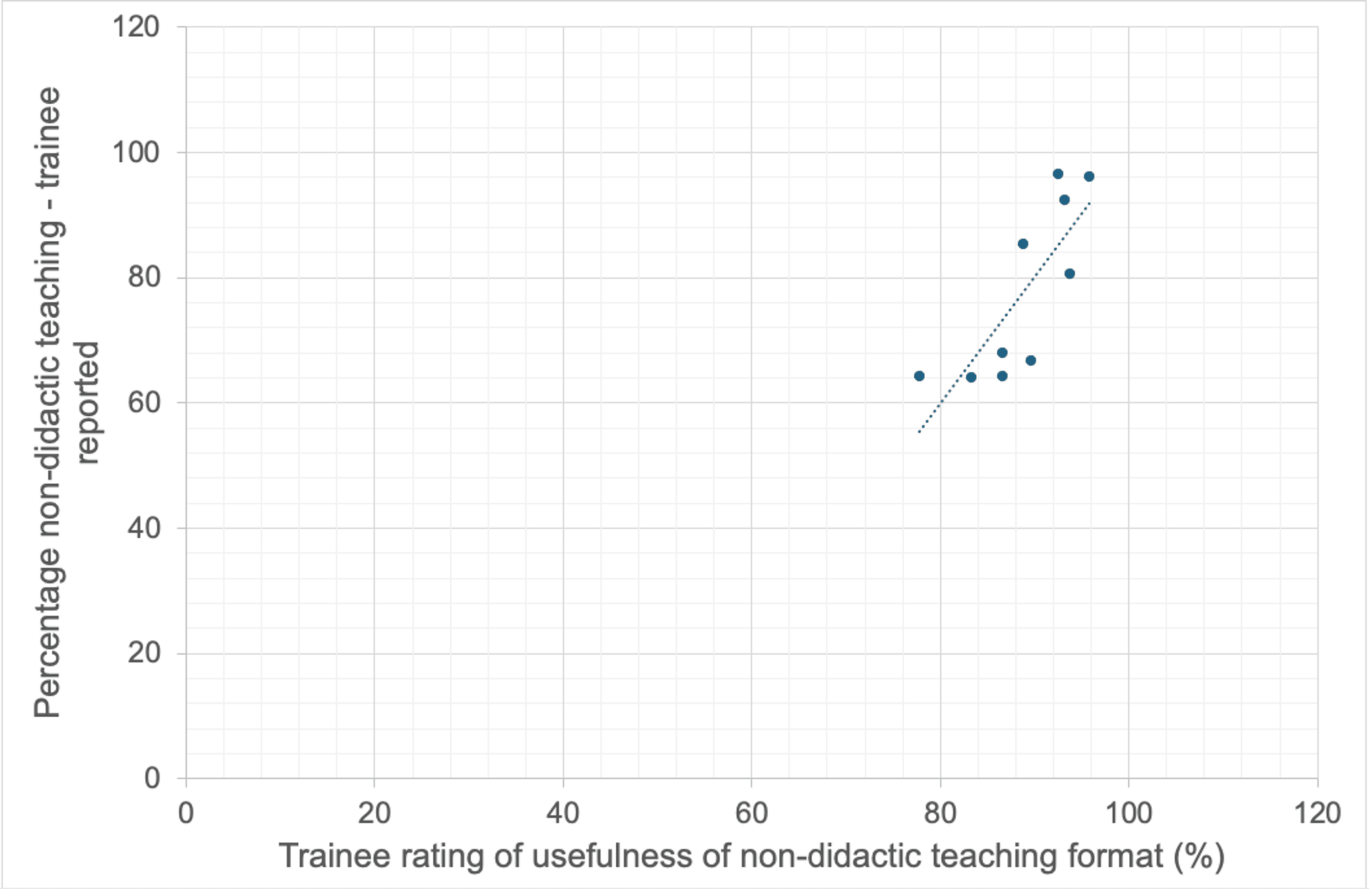
Correlation between trainee rating of non-didactic teaching usefulness and proportion of non-didactic teaching per session.

Following the change to the small group teaching format, trainees were asked to rate the percentage of each session they felt was non-didactic. This correlated positively with the teaching session feedback score, rs = 0.061, p = 0.866 (Spearman’s Rho calculator), and with how useful the trainees rated this style of teaching in comparison to didactic lectures, rs = 0.795, p = 0.005 (Figures 2-3).

## Discussion

The current study assessed the introduction of a small group format for a regional trauma and orthopaedics teaching programme for higher surgical trainees compared to the previous format, which utilized didactic lectures. The most important finding is a positive correlation between the trainee feedback scores and the proportion of the session schedule that was comprised of the small group format. The strongest positive correlation, which reached statistical significance, was between the proportion of small group work as rated by the trainees and how useful trainees rated this style of teaching for their education. To our knowledge, the assessment of small group teaching’s effectiveness in trauma and orthopaedics higher surgical trainee education has not been previously reported.

The findings of the current study correlate in some aspects with previous literature. Regarding teaching in other areas of medicine, Kiran MS and Mestri SC [10] assessed the effectiveness of small group discussions versus didactic lectures for radiological teaching. In that study, 61.1% strongly agreed that group discussions were better, leading the authors to conclude that small group discussions can be a fertile environment for both individual and group development [10]. Ghorbani N et al. [11] investigated the use of a team-based approach to teaching anatomy to undergraduate physiotherapy students compared to didactic lectures. The authors found a significant improvement in both post-teaching test results and student satisfaction with team-based learning compared to didactic lectures [11]. The common finding of increased trainee satisfaction when using a small group format to deliver teaching across a range of subject matter supports the current study’s findings.

Focusing on literature specific to trauma and orthopaedics teaching, Costa ML et al. [7] assessed the use of group discussions versus lectures in teaching undergraduate trauma and orthopaedics. Consistent with the current study, they found that students in the interactive discussion groups rated the teaching significantly higher than those in the lecture group [7]. These findings are consistent with the results of the current study, however, these previous studies did not assess the use of this teaching format in post-graduate or higher surgical training in trauma and orthopaedics, which was a unique factor of our findings.

The available published literature regarding post-graduate surgical teaching is minimal. El-Sayed C et al. [8] collected feedback from general surgery higher trainees on the utilization of virtual learning in their teaching sessions. 91% rated the administration and delivery of the teaching as excellent or very good; however, there was no comparison group, no assessment of the amount of knowledge gained, and this study did not focus on the use of small group teaching [8]. Roels N et al. [9] assessed the use of online case discussions for upper limb extremity trauma surgeons. They showed a significant improvement in knowledge comparing pre- and post-course multiple choice assessments [9]. Including the use of online case discussions has some similarity to the current study, however, they did not focus on face-to-face sessions or other aspects of small group teaching such as viva practice. Interestingly, when asked their level of agreement with the statement ‘When all the technical issues are addressed, online small group discussions can run just as well as in a face-to-face environment,’ only 31% of participants and 10% of faculty strongly agreed [9], which supports the current study’s approach of persevering with in-person teaching.

Despite the well-documented advantages of small group or team-based learning, it also has its limitations. There is an onus on the trainer to reward students for individual and preemptive study as well as provide the trainees with the opportunity to use this knowledge in a meaningful and applicable way [10]. However, the informal trainer feedback we received was that the small group sessions were more enjoyable to deliver and a more effective way of delivering the FRCS curriculum compared to large group didactic lectures.

There are limitations to the current study. We focused on trainee feedback on the sessions but did not assess the knowledge accrued. Pre-existing literature has commented on the ability of teaching methods to influence the amount of knowledge successfully accrued; Tan NC et al. [13] reported team-based learning as an effective technique for improving knowledge of clinical neurology in undergraduates when compared to passive learning, with post-teaching test scores being significantly higher than pre-teaching scores. Vasan NS et al. [5] reviewed the use of team-based learning in teaching anatomy and also found a greater improvement in knowledge gained compared to lecturing. Regarding trauma and orthopaedics teaching, Costa ML et al. [7] found that medical students performed significantly better in the end-of-placement test when using group discussions rather than didactic lectures. Similarly, Bulstrode C et al. [6] found that using small group trainee-directed teaching sessions (colloquially called ‘donut rounds’) with medical students resulted in significantly higher post-course scores in students with lower pre-course knowledge when compared to didactic lectures. The effectiveness of the higher surgical training curriculum teaching is ultimately assessed by the trainees’ performance in the FRCS examination, and it will be interesting to review the success rates for the deanery in future sittings.

Using a variety of trainers across the teaching sessions could also be viewed as a limitation as it adds additional uncontrolled variables with regards to each individual's teaching style. The authors, however, felt that this best represented how higher surgical training teaching is delivered and therefore made the results of this study more applicable to the future programme and its development. We also attempted to minimize the amount of variability across each teaching episode by giving all trainers the same instructions prior to them preparing their session.

A further limitation of this study is potential bias in the feedback received. Whilst the small group format was well received in the current study with a feedback rating of 94.6%, this was only marginally higher than the previous mixed teaching format. This may be due to positive feedback bias with only trainees who feel very strongly about the quality of the teaching completing the feedback. Furthermore, trainees may be reluctant to give negative feedback. Issues with the usefulness of teacher feedback have been previously reported [14]. We tried to combat any apprehension about giving negative feedback by making the forms anonymous. This, however, has its own issues, in that the authors were then unable to analyze data on who was sending feedback and assess whether there were any trainee factors that increased the likelihood of positive or negative ratings. An additional issue with the feedback format was a low response rate. The mean percentage of trainees who sent feedback was only 50%; in the future, the quality and reliability of the feedback may be improved by making its completion mandatory prior to the end of each session.

## Conclusions

Higher surgical training programme was well received by trainees, who perceived this format to be more useful for their training in preparation for the FRCS exam. There was a significant positive correlation between the proportion of small group teaching and how useful the trainees rated this format for their learning. The teaching programme and education directors aim to continue implementing and developing this format, and to measure its effectiveness in terms of knowledge retention and successful completion of the FRCS examination.

## Figures and Tables

**Figure 4:**
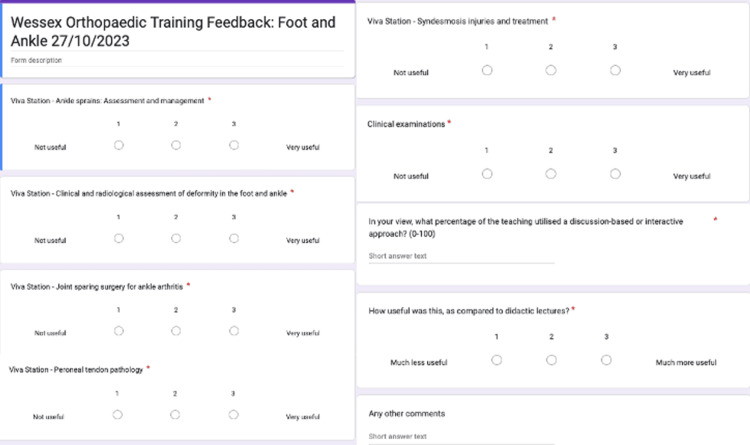
Example of teaching feedback form.

## Appendices

Appendix 1 
